# Association Between Serum Lipids and Survival in Patients With Amyotrophic Lateral Sclerosis: A Meta-analysis and Population-Based Study

**DOI:** 10.1212/WNL.0000000000201657

**Published:** 2023-03-07

**Authors:** Mark R. Janse van Mantgem, Wouter van Rheenen, Anemone V. Hackeng, Michael A. van Es, Jan H. Veldink, Leonard H. van den Berg, Ruben P.A. van Eijk

**Affiliations:** From the Department of Neurology (M.R.J.M., W.R., A.V.H., M.A.E., J.H.V., L.H.B., R.P.A.E.), UMC Utrecht Brain Center, and Biostatistics & Research Support (R.P.A.E.), Julius Center for Health Sciences and Primary Care, University Medical Center Utrecht, The Netherlands.

## Abstract

**Background and Objective:**

To explore the association between lipids, polygenic profile scores (PPS) for biomarkers of lipid metabolism, markers of disease severity, and survival in patients with amyotrophic lateral sclerosis (ALS).

**Methods:**

We meta-analyzed the current literature on the prognostic value of lipids in patients with ALS. Subsequently, we evaluated the relationship between lipid levels at diagnosis, clinical disease stage, and survival in all consecutive patients diagnosed in the Netherlands. We determined the hazard ratio (HR) of each lipid for overall survival, defined as death from any cause. A subset of patients was matched to a previous genome-wide association study; data were used to calculate PPS for biomarkers of lipid metabolism and to determine the association between observed lipid levels at diagnosis and survival.

**Results:**

Meta-analysis of 4 studies indicated that none of the biomarkers of the lipid metabolism were statistically significantly associated with overall survival; there was, however, considerable heterogeneity between study results. Using individual patient data (N = 1,324), we found that increased high-density lipoprotein (HDL) cholesterol was associated with poorer survival (HR of 1.33 (95% CI 1.14–1.55, *p* < 0.001)). The correlation between BMI and HDL cholesterol (Pearson *r* −0.26, 95% CI −0.32 to −0.20) was negative and between BMI and triglycerides (TG) positive (Pearson *r* 0.18, 95% CI 0.12–0.24). Serum concentrations of total cholesterol and LDL cholesterol were lower in more advanced clinical stages (both *p* < 0.001). PPS for biomarkers of lipid metabolism explained 1.2%–13.1% of their variance at diagnosis. None of the PPS was significantly associated with survival (all *p* > 0.50).

**Discussion:**

Lipids may contain valuable information about disease severity and prognosis, but their main value may be driven as a consequence of disease progression. Our results underscore that gaining further insight into lipid metabolism and longitudinal data on serum concentrations of the lipid profile could improve the monitoring of patients and potentially further disentangle ALS pathogenesis.

Lipids act as structural components of neuronal membranes, signaling molecules and energy substrates required for normal functioning of neurons.^1^ Although the exact pathophysiologic mechanisms underlying amyotrophic lateral sclerosis (ALS) are unknown,^2^ it is likely that the origins of the condition lie in a multistep process,^3^ followed by intraneuronal disease propagation, altered neuronal metabolism, and ultimately neuronal death. Dysregulated energy metabolism is a consequence of this process,^4^ which also affects biomarkers of the lipid metabolism, such as cholesterol, its carriers (i.e. LDL and HDL cholesterol), and triglycerides (TG). Albeit little is known about changes in the preclinical stage, 2 recent studies comprising a Mendelian randomized study,^5^ and a prospective cohort study of over 500,000 people,^6^ related premorbid metabolic changes to the risk of ALS.

The association between biomarkers of lipid metabolism, prognosis, and disease progression after disease onset has proven more difficult to characterize. Although high lipid levels have been shown to increase metabolic stress^7-9^ and potentially lead to a more aggressive disease course,^2^ some studies have suggested that abnormal lipid levels may actually be beneficial to the patient's prognosis.^10-14^ Elucidating the interplay between clinical phenotype and lipid metabolism may reveal potential therapeutic interventions and better address the mixed results from dietary interventions obtained thus far.^15,16^ In this study, therefore, we aim to summarize the current literature and to explore the relationships between lipids, ALS survival, polygenic profile scores (PPS) for lipid levels, and markers of disease progression in a large population-based study, to address the disparate data in the literature.

## Methods

A two-step approach was used. First, we conducted a systematic review to summarize and meta-analyze the current literature on the prognostic value of biomarkers of lipid metabolism in patients with ALS. Second, we assessed the prognostic value of lipids in a large population-based cohort study, explored their relationship with disease severity, and assessed the causal association between PPSs and survival after disease onset. Throughout the text, we define “biomarkers of lipid metabolism” as an umbrella term for total cholesterol (TC), low-density lipoprotein cholesterol (LDL-C), high-density lipoprotein cholesterol (HDL-C), and TG.

### Systematic Review

#### Search and Study Selection

We conducted the systematic search in 4 literature databases: PubMed, EMBASE, DARE, and the Cochrane Library; the study protocol for the systematic review is presented in the supplementary material (eAppendix 1: Systematic Review Protocol,, links.lww.com/WNL/C516). Additional forms or information, such as data collection forms, can be provided on request. The primary purpose of the meta-analyses was to provide an explanatory summary of the current literature. All databases were last searched in June 2022. Search terms included the MeSH terms: “Amyotrophic Lateral Sclerosis”; “Motor Neuron Disease”; “Cholesterol”; “Cholesterol, LDL”; “Cholesterol, HDL”; “Triglyceride”; “Lipid”; “Prognosis”; “Survival”; “Mortality”; “Kaplan-Meier estimate”; and “Proportional Hazard Models.” Studies were selected on the basis of the following inclusion criteria: (1) participants diagnosed with ALS according to the revised El Escorial criteria (EEC)^17^; (2) reporting of at least one of the following measurements: TC, HDL-C, LDL-C, or TG, obtained after symptom onset; (3) reporting of survival time and hazard ratio (HR); and (4) written in English or Dutch. Study eligibility was not based on sample size. All articles were screened independently by 2 reviewers for title and abstract (M.J.v.M. and A.H.). Included and excluded articles were discussed; if no consensus was reached, a third reviewer was consulted (R.P.A.v.E.).

#### Data Collection and Meta-analysis

For each included study, we extracted the following variables: author, publication year, country, number of participants, and statistical analysis parameters (that is, covariates, HR, and 95% CI). We used the Quality in Prognosis Studies tool to determine the quality and risk of bias of the included articles.^18^ Studies that provided a HR for at least one, nondichotomized, biomarker of the lipid metabolism were included in the meta-analysis. Standardized HRs (SE) were back-transformed to mmol/L by dividing by the study standard deviation; if studies reported biomarkers of lipid metabolism in mg/dL, data were converted to mmol/L by dividing the HR (SE) by 0.02586 for TC, LDL-C, and HDL-C or by 0.01129 for TG. Meta-analyses were conducted using a Bayesian hierarchical model using a noninformative uniform prior for the log HR and a weakly informative prior for the heterogeneity parameter (half normal with standard deviation of 0.5). As sensitivity analysis, we varied the prior for the heterogeneity parameter using either a standard deviation of 0.25 or 1.0.^19^ Funnel plots were used to visually inspect publication bias and study heterogeneity (eFigure 1, links.lww.com/WNL/C516). We estimated the heterogeneity between studies using the I^2^ statistic and expressed this as percentage. The meta-analyses provide the pooled HR on survival across studies for each biomarker of lipid metabolism in mmol/L.

#### Population-Based Cohort

For the second part of this study, we conducted a prospective analysis of the national registry of the Netherlands ALS Center, selecting all consecutive patients diagnosed in the University Medical Center Utrecht (UMCU), Utrecht, the Netherlands, between January 1, 2012, and December 31, 2017, to ensure sufficient follow-up time for survival. All patients were diagnosed with possible, probable laboratory supported, probable, or definite ALS.^17^ The UMCU is a referral center for all patients with ALS across our country. All clinical characteristics were collected at the time of diagnosis. The King's clinical staging system^20^ was determined according to the standard operating procedures provided by the European Network to Cure ALS (ENCALS).^21^ Patients with more than 30 hexanucleotide repeats in the *C9orf72* gene were considered to be *C9orf72* carriers.^22^ We defined survival time as time between date of diagnosis and date of death or date last known to be alive. Survival information was updated at quarterly intervals by cross-referencing with the municipal population register. All patients were administratively censored on 9 July 2020. Data were further supplemented with the revised ALS functional rating scale (ALSFRS-R) collected at time of diagnosis.^15^ For a subset of patients, longitudinal data of the ALSFRS-R were available, obtained during either clinical follow-up or previous participation in clinical research.

### Blood Sample Collection

Blood samples were collected from patients in a nonfasting state on the day of diagnosis or within one month after diagnosis.^23^ We determined TC, LDL cholesterol, HDL cholesterol, and TG with the Beckman Coulter AU5800 clinical chemistry analyzer series. Normal ranges were defined according to the central diagnostic laboratory of the UMCU: TC 3.5–6.5 mmol/L, LDL-C < 3.5 mmol/L, HDL-C > 0.90 mmol/L for male patients, HDL-C > 1.1 mmol/L for female patients, and TG 0.0–2.0 mmol/L.

### Statistical Analysis

We performed our statistical analyses using RStudio (version 1.1.4, RStudio: Integrated Development for R, Boston, USA, rstudio.com/). Mean and SD were determined and summarized for continuous variables; for categorical variables, we determined frequency and proportion. The Cox proportional hazard model was applied to assess the association between the risk of death and biomarkers of lipid metabolism at diagnosis. All models were adjusted for the 8 clinical predictors—combined in a linear predictor—from the ENCALS survival model,^24^ namely age at onset, diagnostic delay, bulbar onset, definite ALS according to the revised EEC,^17^ prediagnostic progression rate (ΔFRS),^25^ percentage (%) of predicted forced vital capacity, presence of frontotemporal dementia (FTD), and carrier of the *C9orf72* repeat expansion. For each analysis, the following sensitivity analyses were conducted: (1) adding an interaction term between biomarker level and sex (i.e. is the effect of the biomarker different for male patients vs female patients?) and similarly for age at diagnosis, (2) adding quadratic terms to explore potential nonlinear relationships between the risk of death and the biomarker level, and (3) additional adjustment for body mass index (BMI) and weight loss, factors known to be associated with both the lipid level and survival.^26^ Data missing for any variable except the outcome were addressed by creating multiple imputed data sets (n = 100), using predictive mean and bootstrapping, discarding the first 100 iterations (burn-in). In total, 9.2% of all observations were missing and, therefore, imputed. All covariates were included in a stratified imputation model per diagnostic year; survival time was included as cumulative hazard rate (Nelson-Aalen estimator).^27^ The results across imputations were pooled using Rubin rules.^28^

We further explored longitudinal trends in disease progression rate by assessing the relationship between lipid levels at diagnosis and decrease in ALSFRS-R since diagnosis using linear mixed-effects models. Models contained a fixed effect for time since diagnosis (in months), lipid level, and the interaction between time and lipid level; the random part contained a random slope for time and intercept per patient. We used a likelihood ratio test to assess the significance of the interaction between lipid level and time (i.e., is the rate of ALSFRS-R progression dependent on lipid level?). In addition, we assessed the cross-sectional association between lipid levels, BMI, and King's Clinical Staging^20^ at diagnosis using linear regression models. Sensitivity analyses were conducted by introducing interaction terms for sex to assess potential male-female differences. All analyses of the TG level were performed on the natural logarithm scale because of their right-skewed distribution.

### PPS

As an exploratory analysis, we estimated PPS for biomarkers of lipid metabolism.^29^ The PPS estimates the sum of additive genetic effects across all alleles that affect the biomarkers of lipid metabolism at the patient level. We used the PPS to explore a potential genetic link between lipid metabolism, ALS, and survival time by assessing (1) how much of the variance in biomarker levels at diagnosis can be explained by genetic profile scores and (2) whether the genetic profile score itself is associated with overall survival time. Because PPS does not change over time,^30^ a statistical association between the genetic profile score and survival may be evidence of abnormal lipid levels caused by genetic variation or hold potential for therapeutic interventions.^30^ Moreover, their time invariance allowed us to estimate the link between the genetic profile score and overall survival time, defined as time between symptom onset and death.

For all individuals who were enrolled in both our population-based registry and our latest genome-wide association study (GWAS),^5^ we calculated the PPS. PPS was based on summary statistics from a GWAS on biomarker levels of lipid metabolism in the UK Biobank.^31^ For each single-nucleotide polymorphism, we calculated a weight for each biomarker using the summary-BavesR module in the Genome-Wide Complex Trait Bayesian analysis toolkit (default parameters)^29^ and a linkage-disequilibrium matrix originating from 50,000 unrelated individuals of inferred European ancestries included in the UK Biobank. Because the genotype data originated from several different cohorts in the ALS GWAS, we scaled the PPS per GWAS cohort to a mean of zero and a standard deviation of 1. Linear regression models were used to calculate how much of the variance in the biomarker level was explained by their PPS (expressed as adjusted R^2^); 95% confidence intervals were obtained by means of bootstrapping. Simple univariable Cox models for overall survival time (i.e., from onset to death) were used to estimate HRs.

### Standard Protocol Approvals, Registrations, and Patient Consents

The medical ethics committee and institutional review board of the University Medical Center Utrecht (METC NedMec) approved this study (Study Registration Number: METC 19–190). Written consent was obtained from all study participants before this study.

### Data Availability

All protocol, analyses, and anonymized data will be shared on request. We take full responsibility for data, analyses and interpretation, and conduct of the research.

## Results

### Systematic Review and Meta-analysis

**Figure 1:**
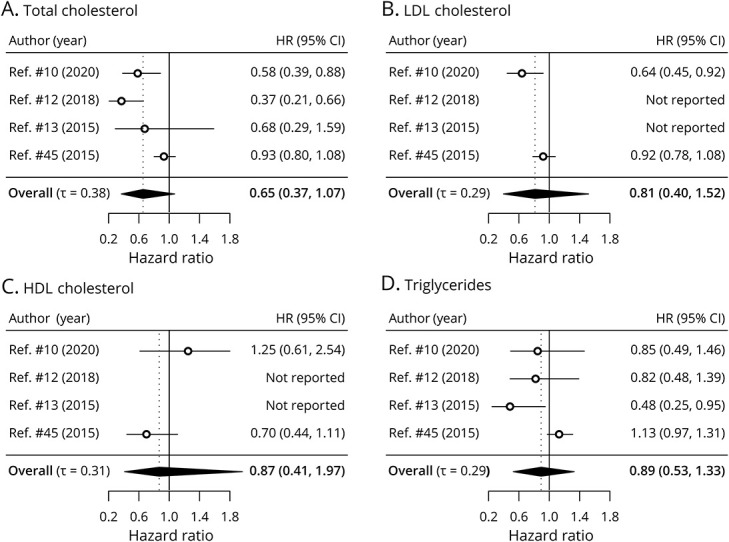
Forest Plot of the Included Studies for Biomarkers of Lipid Metabolism Meta-analysis of the reported HRs in the literature. HR of each lipid for survival defined as the time in months from study enrollment to death from any cause or administrative censoring. The overall HR reflects the pooled HR across studies in mmol/L. Abbreviations: HR = hazard ratio; CI = credible interval.

Of the 624 citations screened, 9 articles were included (eFigure 2, links.lww.com/WNL/C516), 5 of which found a significant association between survival time and serum levels of TC, LDL/HDL ratio, HDL cholesterol, or TG; their characteristics are summarized in Table 1. Studies included different prognosticators in their multivariable model; none adjusted for all known prognosticators in patients with ALS.^24^ 4 studies reported a nondichotomized HR and were included in the meta-analysis, resulting in a total sample size of 1,120 patients (Figure 1). The risk of bias assessment of the individual studies is presented in eFigure 3. None of the biomarkers of the lipid metabolism reached statistical significance (Figure 1), although the 95% credible intervals included clinically relevant effect sizes. There was, however, considerable heterogeneity between study results, reflected as τ, indicating possible differences in methodology. Changing the prior assumptions resulted in similar findings (not shown). In eFigure 1, we provide the funnel plot to explore publication bias; it should be noted that, given the small number of studies, their interpretation is limited.

**Table 1:**
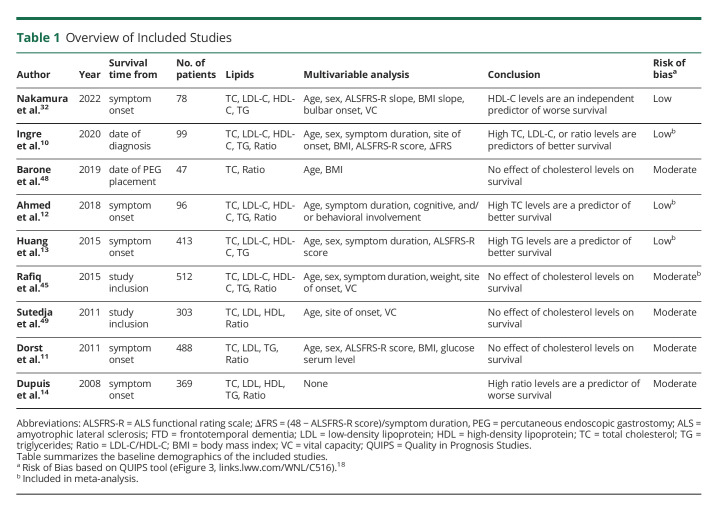
Overview of Included Studies

### Population-Based Cohort

In total, 1,324 patients with ALS were enrolled in our population-based registry. At the time of administrative censoring (July 2020), 1,185 deaths (89.5% of enrolled population) had occurred during 2,370 person-years of follow-up. The median survival since diagnosis was 16.5 months (95% CI 15.7–17.5). Baseline characteristics of the cohort are listed in Table 2; 688 patients (52%) had been enrolled in our latest GWAS study and were included in the PPS analysis. Overall, 20.1% of the patients had elevated TC, 42.0% elevated LDL-C, 4.9% reduced HDL-C, and 19.2% elevated TG levels on the day of diagnosis.

**Table 2:**
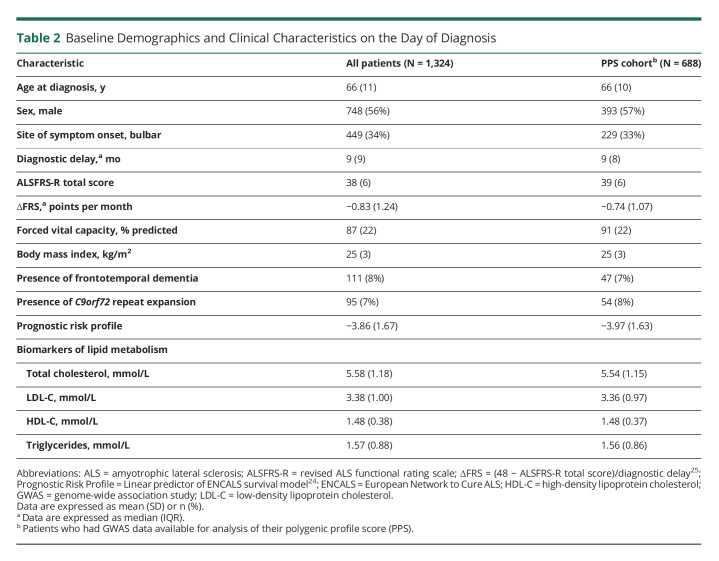
Baseline Demographics and Clinical Characteristics on the Day of Diagnosis

After adjustment for age, site of onset, diagnostic delay, prediagnostic progression rate (∆FRS), vital capacity, presence of FTD, *C9orf72* repeated expansion, and El Escorial classification,^24^ a 1 mmol/L increase of HDL-C was found to be associated with a higher risk of death and shorter survival time after ALS diagnosis, HR of 1.33 (95% CI 1.14–1.55, *p* < 0.001, Table 3). This effect was larger for male patients than for female patients: HR (male patients) 1.48 vs HR (female patients) 1.13, although not statistically significantly different (interaction term *p* = 0.094). The effect was similar for different ages at diagnosis (HR-interaction 1.00; 95% CI 0.98–1.02, *p* = 0.97). Introduction of a nonlinear term did not result in a significant model improvement (*p* = 0.84). Additional adjustment for weight loss (HR of 1.37, 95% CI 1.17–1.61) or body mass index (HR of 1.28, (95% CI 1.09–1.50) did not alter our results.

**Table 3:**
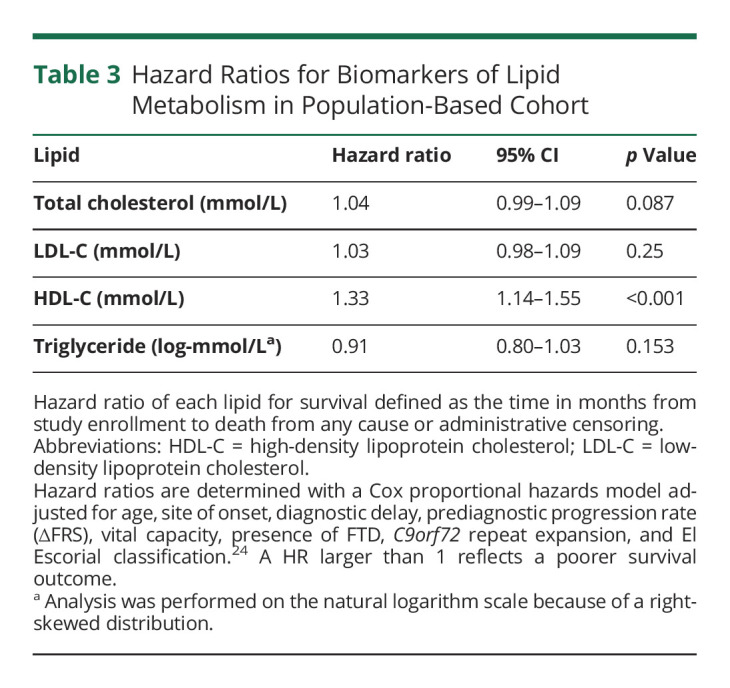
Hazard Ratios for Biomarkers of Lipid Metabolism in Population-Based Cohort

Longitudinal ALSFRS-R data, that is, 2 or more measurements, were available for 419 of the 1,324 patients (31.6%). Average progression rate after diagnosis was 0.79 points per month (95% CI 0.73–0.85). With each mmol/L increase in HDL-C, the monthly ALSFRS-R progression rate increased by 0.10 points per month (95% CI −0.07 to 0.26, *p* = 0.21), indicating a similar directional effect as observed on survival, albeit not statistically significant. None of the other biomarkers of the lipid metabolism was significantly associated with the monthly progression rate (all *p* > 0.15).

**Figure 2:**
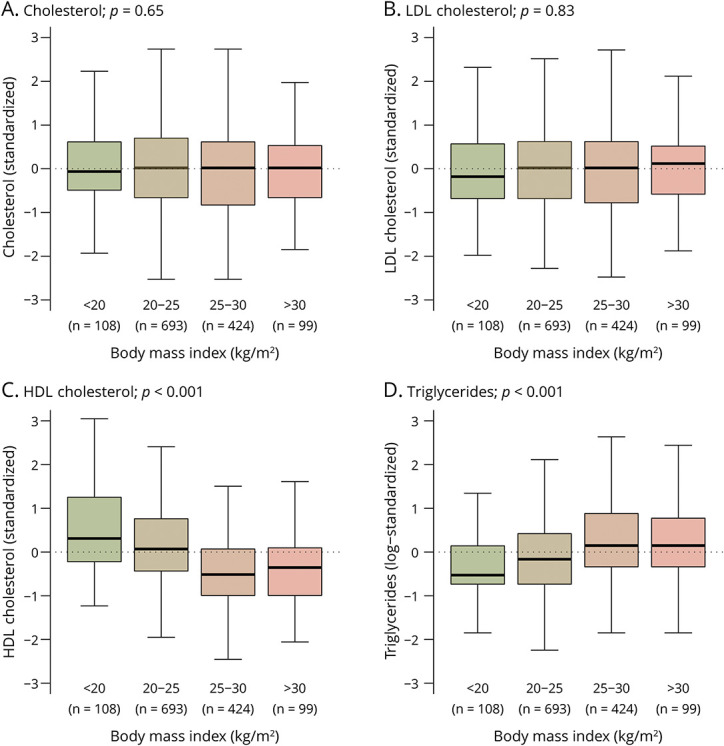
Biomarkers of the Lipid Metabolism Stratified by BMI Category at Diagnosis Boxplots summarizing the cross-sectional concentrations of the lipids linked to body mass index (BMI) at diagnosis. Scales are standardized to provide a direct comparison between lipids; interpretation is straightforward, where the scale reflects the number of standard deviations above or below the mean lipid level as presented in Table 2. Abbreviations: HDL = high-density lipoprotein; LDL = low-density lipoprotein. *p* values are based on the likelihood ratio test.

**Figure 3:**
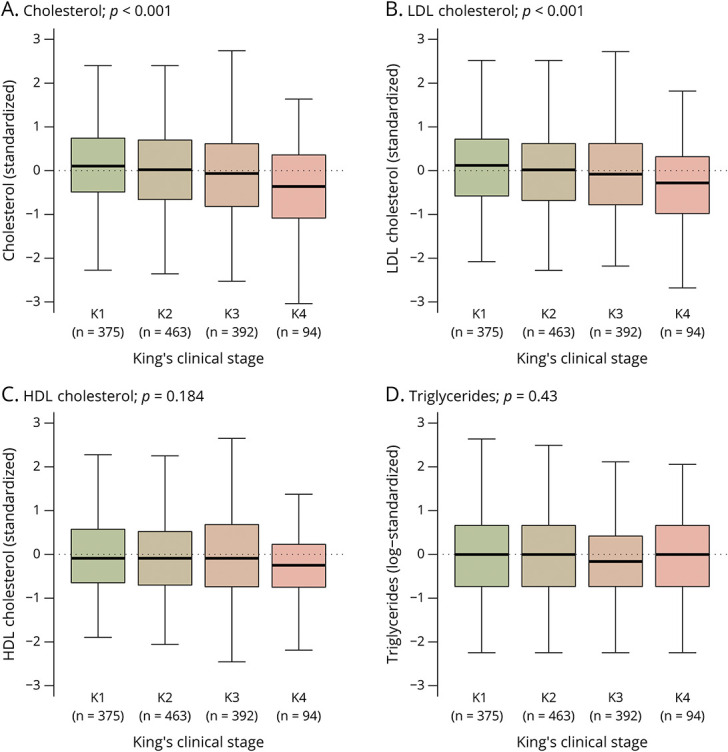
Biomarkers of the Lipid Metabolism Stratified by King's Clinical Staging at Diagnosis Boxplots summarizing the cross-sectional concentrations of the lipids linked to the 4 King's clinical stages. Scales are standardized to provide a direct comparison between lipids; interpretation is straightforward, where the scale reflects the number of standard deviations above or below the mean lipid level as presented in Table 2. Abbreviations: HDL = high-density lipoprotein; LDL = low-density lipoprotein. *p* values are based on the likelihood ratio test.

Figure 2 and Figure 3 present the standardized distributions of the biomarkers of lipid metabolism stratified by BMI category and King's clinical stage at diagnosis, respectively. Both HDL-C (Pearson *r* −0.26, 95% CI −0.32 to −0.20) and TG (Pearson *r* 0.18, 95% CI 0.12–0.24) were associated with BMI at diagnosis (both *p* < 0.001); these relationships were similar for male patients and female patients (both interaction terms *p* > 0.40). Similarly, TC and LDL-C depended on King's clinical staging and showed a declining trend for more advanced disease stages (both *p* < 0.001); again, these associations were similar for male patients and female patients (both interaction terms *p >* 0.75). The results were similar when categorizing the ALSFRS-R into 4 equal categories (*results not shown*).

### Analysis of PPSs

Finally, in Table 4, we summarize how much of the variance in lipid levels observed at diagnosis can be attributed to the respective PPS, expressed as adjusted R^2^, and how the PPS relate to overall survival since symptom onset. Each PPS was significantly correlated with the respective lipid level (Pearson *r*_*TC*_ 0.11, *p* = 0.002; Pearson *r*_*LDL-C*_ 0.23, *p* < 0.001; Pearson *r*_*HDL-C*_ 0.36, *p* < 0.001; Pearson *r*_*log-TG*_ 0.33, *p* < 0.001), with the explained variance at diagnosis ranging from 1.2% to 13.1%. None of the PPS was, however, significantly associated with overall survival time (all *p* > 0.50).

**Table 4:**
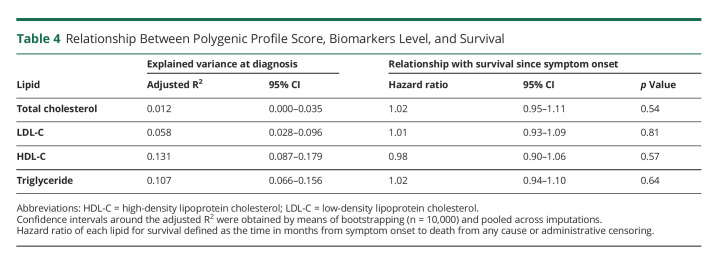
Relationship Between Polygenic Profile Score, Biomarkers Level, and Survival

## Discussion

In this study, we have shown the extensive variability in the literature regarding the prognostic value of the lipid profile. The study heterogeneity is mainly driven by differences in study design, statistical models, sample size, and the patient population enrolled. In the second part of our study, only HDL cholesterol had additional prognostic value for predicting survival after diagnosis in patients with ALS in a prospective, population-based registry. Changes in components of the lipid profile were primarily related to disease severity. We found no immediate associations, however, between lipid-based polygenic scores and overall survival, yet another indication that changes in the lipid profile may be primarily a consequence of disease. Our results underscore that obtaining greater insight into lipid metabolism and longitudinal data on serum concentrations of the lipid profile could improve the monitoring of patients and potentially further disentangle ALS pathogenesis.

First, our literature search into the relationship between survival and lipid profile showed that the results of these studies are mixed.^10-14,32^ The included studies analyzed lipids either continuously or as binary factor (e.g., high vs low). Binary categorization of the lipid levels into normal or abnormal may lead to spurious associations and be too limited to describe the gradual associations with prognosis. When pooling results across studies in a meta-analysis, none of the lipids were statistically significantly associated with survival, but individual study results varied considerably. The variation may be explained by (1) differences in the disease stage and the phenotype of the population enrolled and (2) differences in study methodology (e.g., follow-up time, statistical approach, and sample size).

Second, our analysis of a population-based registry confirmed the nonprognostic value of most lipids; HDL-C was, however, found to be predictive of overall survival since diagnosis. This finding was recently confirmed in both Japanese^32^ and Swedish^10^ patients, although insignificantly in the latter. We were not able to show the association between HDL-C and disease progression determined by the ALSFRS-R, as follow-up data were limited. The prognostic value of HDL-C could be the result of a surrogate association with disease progression. Respiratory insufficiency or symptoms of dyspnea have been associated with the lipid profile,^33^ while dietary changes alter lipid concentrations.^34^ Weight loss is observed in up to 60% of patients with ALS,^26^ and changes in BMI have a direct impact on the lipid profile.^35,36^ This impact was also found in our study population; there was a strong association between HDL-C and BMI, where HDL-C increases as BMI decreases. However, adjusting for BMI or other markers of disease severity minimally affected the association between HDL-C and survival. Albeit speculative, one could also hypothesize that the prognostic association might partially reflect a premanifest or prodromal sign of ALS. For example, production of oxidized derivatives of excess cholesterol might be caused by deficiencies in cholesterol metabolism,^7^ which in turn may induce neuronal damage leading to muscle function loss.^7,37^ Deficiencies in cholesterol metabolism may also lead to dysregulated transport of cholesterol and result in toxicity in the brain.^38^

In an attempt to disentangle this potential causality between lipids and survival, we estimated PPS for biomarkers of lipid metabolism to explore genetic links with lipid metabolism and ALS survival time. As PPS does not change over time,^30^ any association between PPS and survival may be an indication that premorbid changes in lipids result in a more aggressive disease as expressed in overall survival time.^29^ Our results highlight the predictive value and utility of PPS in patients with ALS as surrogate for actual lipid levels but also underscore that over 80% of the variance in the actual lipid levels were not captured by the PPS. Taking into account, the absence of a large effect between PPS and survival time and the results from other studies in which PPSs were more predictive for actual lipid levels,^39^ these observations may support reverse causality, where lipid levels change as a consequence of the disease rather than vice versa.

The clinical relevance of these observations depends on the setting and the intended use of the PPS. Despite the large sample size of our cohort, we were primarily powered to detect HRs of 1.1 or greater. An HR of 1.1 would translate to a 46.4% difference in hazard when comparing a patient with -2SD (∼2.5th percentile) vs a patient with +2SD (∼97.5th percentile). Smaller effect sizes, therefore, could still be deemed relevant, although detecting, for example, an HR of 1.05 or greater with 90% power would require approximately 4,500 survival events. Larger GWASs that link overall survival time to PPS may, therefore, be needed to further investigate potential causal or etiological relationships.^30^ Moreover, determining whether a change in the lipid level precedes a change in clinical progression requires longitudinal observations with repeated blood samples to provide more definite evidence.^40^ In such studies, it would be key to carefully collect other parameters that influence lipids, which were not collected in our study, such as smoking,^41^ diet or the use of cholesterol-lowering drugs (CLD),^42^ and preferably assess serum concentration in a fasting state to minimize variability.^43,44^ Finally, 42.0% of our patient population had elevated serum concentrations of LDL-C; the mean serum HDL-C was comparable with that of the general Dutch population.^43^ Studies that enrolled patients with ALS have reported similar serum concentrations.^10,45^ HDL-C values were more or less the same as those found in the general population; however, an elevated LDL-C can be found in approximately 50%–60% of people of similar age in the Netherlands.^46,47^ Patients with ALS, therefore, may have lower levels of LDL-C compared with the general population,^46^ supporting our finding of decreasing levels in more advanced disease stages. Enrollment of a more geographically and culturally diverse population may improve generalizability of the exact association between lipids and overall survival in ALS, but dedicated case-control studies are needed to confirm true differences in lipid levels between patients with ALS and the general population. Moreover, although our study indicates a relationship with cross-sectional clinical stages, determining whether a change in the lipid level precedes a change in clinical progression requires longitudinal observations with repeated blood samples to provide more definite evidence.

In conclusion, lipids may contain valuable information about disease severity and prognosis because serum concentrations seem to be dependent on disease severity. Our results underscore that gaining further insight into lipid metabolism and longitudinal data on serum concentrations of the lipid profile could improve the monitoring of patients. Because our results are not in line with previous studies on a causal effect of the lipid profile on ALS disease progression, we believe that this new information may contribute to ongoing efforts to disentangle ALS pathogenesis.

## Glossary

ALS: amyotrophic lateral sclerosis
ALSFRS: ALS functional rating scale
BMI: body mass index
EEC: El Escorial criteria
ENCALS: European Network to Cure ALS
FTD: frontotemporal dementia
HDL: high-density lipoprotein
HDL-C: HDL cholesterol
HR: hazard ratio
LDL-C: low-density lipoprotein cholesterol
PPS: polygenic profile score
TC: total cholesterol
TG: triglycerides

## References

[1] Tracey TJ, Kirk SE, Steyn FJ, Ngo ST. The role of lipids in the central nervous system and their pathological implications in amyotrophic lateral sclerosis. Semin Cell Dev Biol. 2021;112:69-81. 32962914 10.1016/j.semcdb.2020.08.012

[2] Cunha-Oliveira T, Montezinho L, Mendes C, et al. Oxidative stress in amyotrophic lateral sclerosis: pathophysiology and opportunities for pharmacological intervention. Oxid Med Cell Longev. 2020;2020:1-29. 10.1155/2020/5021694 PMC7683149 33274002

[3] Al-Chalabi A, Calvo A, Chio A, et al. Analysis of amyotrophic lateral sclerosis as a multistep process: a population-based modelling study. Lancet Neurol. 2014;13(11):1108-1113. 25300936 10.1016/S1474-4422(14)70219-4 PMC4197338

[4] Guillot SJ, Bolborea M, Dupuis L. Dysregulation of energy homeostasis in amyotrophic lateral sclerosis. Curr Opin Neurol. 2021;34(5):773-780. 34343139 10.1097/WCO.0000000000000982

[5] van Rheenen W, van der Spek RA, Bakker MK, et al. Common and rare variant association analyses in Amyotrophic Lateral Sclerosis identify 15 risk loci with distinct genetic architectures and neuron-specific biology. Nat Genet. 2021;53:1636-1648. 34873335 10.1038/s41588-021-00973-1 PMC8648564

[6] Thompson AG, Talbot K, Turner MR. Higher blood high density lipoprotein and apolipoprotein A1 levels are associated with reduced risk of developing amyotrophic lateral sclerosis. J Neurol Neurosurg Psychiatry 2021;0:1-7. 10.1136/jnnp-2021-327133 PMC8685635 34518331

[7] Abdel-Khalik J, Yutuc E, Crick PJ, et al. Defective cholesterol metabolism in amyotrophic lateral sclerosis. J Lipid Res. 2017;58(1):267-278. 27811233 10.1194/jlr.P071639 PMC5234729

[8] Cutler RG, Pedersen WA, Camandola S, Rothstein JD. Evidence that accumulation of ceramides and cholesterol esters mediates oxidative stress-induced death of motor neurons in amyotrophic lateral sclerosis. Ann Neurol. 2002;52(4):448-457. 12325074 10.1002/ana.10312

[9] Wang Z, Bai Z, Qin X, Cheng Y. Aberrations in oxidative stress markers in amyotrophic lateral sclerosis: a systematic review and meta-analysis. Oxid Med Cell Longev. 2019;2019:1-9. 10.1155/2019/1712323 PMC6590548 31281567

[10] Ingre C, Chen L, Zhan Y, Termorshuizen J, Yin L, Fang F. Lipids, apolipoproteins, and prognosis of amyotrophic lateral sclerosis. Neurology. 2020;94(17):1-10. 10.1212/WNL.0000000000009322 PMC7274849 32221024

[11] Dorst J, Kühnlein P, Hendrich C, Kassubek J, Sperfeld AD, Ludolph AC. Patients with elevated triglyceride and cholesterol serum levels have a prolonged survival in amyotrophic lateral sclerosis. J Neurol. 2011;258(4):613-617. 21128082 10.1007/s00415-010-5805-z

[12] Ahmed RM, Highton-Williamson E, Caga J, et al. Lipid metabolism and survival across the frontotemporal dementia-amyotrophic lateral sclerosis spectrum: relationships to eating behavior and cognition. J Alzheimer’s Dis. 2018;61(2):773-783. 29254092 10.3233/JAD-170660

[13] Huang R, Guo X, Chen X, et al. The serum lipid profiles of amyotrophic lateral sclerosis patients: a study from south-west China and a meta-analysis. Amyotroph Lateral Scler Front Degener. 2015;16(5‐6):359-365. 10.3109/21678421.2015.1047454 26121273

[14] Dupuis L, Corcia P, Fergani A, et al. Dyslipidemia is a protective factor in amyotrophic lateral sclerosis. Neurology. 2008;70(13):1004-1009. 18199832 10.1212/01.wnl.0000285080.70324.27

[15] Ludolph AC, Dorst J, Dreyhaupt J, et al. Effect of high-caloric nutrition on survival in amyotrophic lateral sclerosis. Ann Neurol. 2020;87(2):206-216. 31849093 10.1002/ana.25661

[16] Wills A-M, Hubbard J, Macklin EA, et al. Hypercaloric enteral nutrition in patients with amyotrophic lateral sclerosis: a randomised, double-blind, placebo-controlled phase 2 trial. Lancet. 2014;383(9934):2065-2072. 24582471 10.1016/S0140-6736(14)60222-1 PMC4176708

[17] Brooks BR, Miller RG, Swash M, Munsat TL, World federation of neurology research group on motor neuron diseases. El escorial revisited: revised criteria for the diagnosis of amyotrophic lateral sclerosis. Amyotroph Lateral Scler. 2000;1(5):293-299. 10.1080/146608200300079536 11464847

[18] Hayden JA, van der Windt DA, Cartwright JL, Co P. Assessing bias in studies of prognostic factors. Ann Intern Med. 2013;158(4):280-286. 23420236 10.7326/0003-4819-158-4-201302190-00009

[19] Röver C, Bender R, Dias S, et al. On weakly informative prior distributions for the heterogeneity parameter in Bayesian random-effects meta-analysis. Res Synth Methods. 2021;12(4):448-474. 33486828 10.1002/jrsm.1475

[20] Roche JC, Rojas-Garcia R, Scott KM, et al. A proposed staging system for amyotrophic lateral sclerosis. Brain. 2012;135(3):847-852. 22271664 10.1093/brain/awr351 PMC3286327

[21] Network to Cure ALS (ENCALS) E. King's ALS Staging SOP. 2014; Accessed May 1, 2021.

[22] Cooper-Knock J, Hewitt C, Highley JR, et al. Clinico-pathological features in amyotrophic lateral sclerosis with expansions in C9ORF72. Brain. 2012;135(3):751-764. 22366792 10.1093/brain/awr365 PMC3286332

[23] Craig SR, Amin RV, Russell DW, Paradise NF. Blood cholesterol screening: influence of fasting state on cholesterol results and management decisions. J Gen Intern Med. 2000;15(6):395-399. 10886474 10.1046/j.1525-1497.2000.03509.x PMC1495473

[24] Westeneng HJ, Debray TPA, Visser AE, et al. Prognosis for patients with amyotrophic lateral sclerosis: development and validation of a personalised prediction model. Lancet Neurol. 2018;17(5):423-433. 29598923 10.1016/S1474-4422(18)30089-9

[25] Kimura F, Fujimura C, Ishida S, et al. Progression rate of ALSFRS-R at time of diagnosis predicts survival time in ALS. Neurology. 2006;66(2):265-267. 16434671 10.1212/01.wnl.0000194316.91908.8a

[26] Janse Van Mantgem MR, Van Eijk RPA, Van Der Burgh HK, et al. Prognostic value of weight loss in patients with amyotrophic lateral sclerosis: a population-based study. J Neurol Neurosurg Psychiatry. 2020;91(8):867-875. 32576612 10.1136/jnnp-2020-322909

[27] White I, Royston P. Imputing missing covariate values for the Coxmodel. Stat Med. 2009;28(10):1982-1998. 19452569 10.1002/sim.3618 PMC2998703

[28] Rubin D, Schenker N. Multiple imputation in health-care databases: an overview and some applications. Stat Med. 1991;10(4):585-598. 2057657 10.1002/sim.4780100410

[29] Wray NR, Lin T, Austin J, et al. From basic science to clinical application of polygenic risk scores: a primer. JAMA Psychiatry. 2021;78(1):101-109. 32997097 10.1001/jamapsychiatry.2020.3049

[30] Meisner A, Kundu P, Zhang YD, et al. Combined utility of 25 disease and risk factor polygenic risk scores for stratifying risk of all-cause mortality. Am J Hum Genet. 2020;107(3):418-431. 32758451 10.1016/j.ajhg.2020.07.002 PMC7477009

[31] Richardson TG, Sanderson E, Palmerid TM, et al. Evaluating the relationship between circulating lipoprotein lipids and apolipoproteins with risk of coronary heart disease: a multivariable Mendelian randomisation analysis. PLoS Med. 2020;17(3):1-22. 10.1371/journal.pmed.1003062 PMC7089422 32203549

[32] Nakamura R, Kurihara M, Ogawa N, et al. Investigation of the prognostic predictive value of serum lipid profiles in amyotrophic lateral sclerosis: roles of sex and hypermetabolism. Sci Rep. 2022;12(1):1-10. 35115598 10.1038/s41598-022-05714-w PMC8814149

[33] Chio A, Calvo A, Ilardi A, et al. Lower serum lipid levels are related to respiratory impairment in patients with ALS. Neurology. 2009;73(20):1681-1685. 19917991 10.1212/WNL.0b013e3181c1df1e

[34] Schwingshackl L, Hoffmann G. Comparison of effects of long-term low-fat vs high-fat diets on blood lipid levels in overweight orObese patients: a systematic review and meta-analysis. J Acad Nutr Diet. 2013;113(12):1640-1661. 24139973 10.1016/j.jand.2013.07.010

[35] Poobalan A, Aucott L, Smith WCS, Avenell A, Jung R, Broom J. Effects of weight loss in overweight/obese individuals and long-term hypertension outcomes: a systematic review. Obes Rev. 2004;5:43-50. 14969506 10.1111/j.1467-789x.2004.00127.x

[36] Shamai L, Lurix E, Shen M, et al. Association of body mass index and lipid profiles: evaluation of a broad spectrum of body mass index patients including the morbidly obese. Obes Surg. 2011;21(1):42-47. 20563664 10.1007/s11695-010-0170-7

[37] Bigini P, Steffensen KR, Ferrario A, et al. Neuropathologic and biochemical changes during disease progression in liver x receptor β-/-mice, a model of adult neuron disease. J Neuropathol Exp Neurol. 2010;69(6):593-605. 20467332 10.1097/NEN.0b013e3181df20e1

[38] De Aguilar JLG. Lipid biomarkers for amyotrophic lateral sclerosis. Front Neurol. 2019;10(APR):1-6. 31019485 10.3389/fneur.2019.00284 PMC6458258

[39] Ye Y, Chen X, Han J, Jiang W, Natarajan P, Zhao H. Interactions between enhanced polygenic risk scores and lifestyle for cardiovascular disease, diabetes, and lipid levels. Circ Genomic Precis Med. 2021;14(1):1-11. 10.1161/CIRCGEN.120.003128 PMC7887077 33433237

[40] Holdom CJ, Janse van Mantgem MR, van Eijk RPA, et al. Venous creatinine as a biomarker for loss of fat-free mass and disease progression in patients with amyotrophic lateral sclerosis. Eur J Neurol. 2021;28(11):3615-3625. 34216521 10.1111/ene.15003

[41] Craig W, Palomaki G, Haddow J. Cigarette smoking and serum lipid and lipoprotein concentrations: an analysis of published data. BMJ. 1989;298(6676):784-788. 2496857 10.1136/bmj.298.6676.784 PMC1836079

[42] Kakafika A, Athyros V, Tziomalos K, Karagiannis A, Mikhailidis D. High density lipoprotein cholesterol and statin trials. Curr Med Chem. 2008;15(22):2265-2270. 18781948 10.2174/092986708785747481

[43] Balder JW, de Vries JK, Nolte IM, Lansberg PJ, Kuivenhoven JA, Kamphuisen PW. Lipid and lipoprotein reference values from 133,450 Dutch Lifelines participants: age- and gender-specific baseline lipid values and percentiles. J Clin Lipidol. 2017;11(4):1055-1064. 28697983 10.1016/j.jacl.2017.05.007

[44] Nordestgaard BG, Langsted A, Mora S, et al. Fasting is not routinely required for determination of a lipid profile: clinical and laboratory implications including flagging at desirable concentration cut-points - a joint consensus statement from the European Atherosclerosis Society and European Fede. Eur Heart J. 2016;37(25):1944-1958. 27122601 10.1093/eurheartj/ehw152 PMC4929379

[45] Rafiq MK, Lee E, Bradburn M, Mcdermott CJ, Shaw PJ. Effect of lipid profile on prognosis in the patients with amyotrophic lateral sclerosis: insights from the olesoxime clinical trial. Amyotroph Lateral Scler Front Degener. 2015;16(7‐8):478-484. 10.3109/21678421.2015.1062517 26161993

[46] Nurmohamed NS, Collard D, Balder JW, Kuivenhoven JA, Stroes ESG, Reeskamp LF. From evidence to practice: development of web-based Dutch lipid reference values. Neth Hear. J. 2021;29(9):441-450. 10.1007/s12471-021-01562-x PMC8397806 33844162

[47] Lipid Tools. 2022 https://www.lipidtools.com/calculator-pages/reference/

[48] Barone M, Viggiani MT, Introna A, et al. Nutritional prognostic factors for survival in amyotrophic lateral sclerosis patients undergone percutaneous endoscopic gastrostomy placement. Amyotroph Lateral Scler Front Degener. 2019;20(7‐8):490-496. 10.1080/21678421.2019.1643374 31347407

[49] Sutedja NA, van der Schouw YT, Fischer K, et al. Beneficial vascular risk profile is associated with amyotrophic lateral sclerosis. J Neurol Neurosurg Psychiatry. 2011;82(6):638-642. 21471184 10.1136/jnnp.2010.236752

